# Dynamic Phenotypic Clustering in Noisy Ecosystems

**DOI:** 10.1371/journal.pcbi.1002017

**Published:** 2011-03-17

**Authors:** Morten Ernebjerg, Roy Kishony

**Affiliations:** 1 Department of Systems Biology, Harvard Medical School, Boston, Massachusetts, United States of America; 2 School of Engineering and Applied Sciences, Harvard University, Cambridge, Massachusetts, United States of America; University of California, Los Angeles, United States of America

## Abstract

In natural ecosystems, hundreds of species typically share the same environment and are connected by a dense network of interactions such as predation or competition for resources. Much is known about how fixed ecological niches can determine species abundances in such systems, but far less attention has been paid to patterns of abundances in randomly varying environments. Here, we study this question in a simple model of competition between many species in a patchy ecosystem with randomly fluctuating environmental conditions. Paradoxically, we find that introducing noise can actually induce ordered patterns of abundance-fluctuations, leading to a distinct periodic variation in the correlations between species as a function of the phenotypic distance between them; here, difference in growth rate. This is further accompanied by the formation of discrete, dynamic clusters of abundant species along this otherwise continuous phenotypic axis. These ordered patterns depend on the collective behavior of many species; they disappear when only individual or pairs of species are considered in isolation. We show that they arise from a balance between the tendency of shared environmental noise to synchronize species abundances and the tendency for competition among species to make them fluctuate out of step. Our results demonstrate that in highly interconnected ecosystems, noise can act as an ordering force, dynamically generating ecological patterns even in environments lacking explicit niches.

## Author Summary

In natural ecosystems, hundreds of species with different characteristics typically live side by side, some competing for the same foods and some preying on others. A central question in ecology is how the abundance of a given species in such an ecosystem depends on its particular characteristics (its phenotype). Clearly, fixed environments can favor certain phenotypes (thick fur in a cold climate), but what happens when environmental conditions fluctuate randomly as e.g. the weather does? We investigated this question using a simple mathematical model of an ecosystem with many competing species. We found that, paradoxically, randomness in the environment can lead to the appearance of ordered clusters of abundant species with similar phenotypes, with the species adopting intermediate phenotypes being much less abundant (a mountains-and-valleys pattern). The clusters move around so that different phenotypes are favored at different times. We found that these effects arise from the tension between the tendency of noise to level out difference in abundances and the tendency of competition to create larger abundance differences.

## Introduction

Species abundances and their variation over time are quantities of fundamental importance in any ecosystem: understanding the forces that shape them is a key part of central problems in ecology, ranging from conceptual questions about the role of neutral processes [1], [2] to practical issues in biodiversity conservation [3]. One major driver of changes in species abundances is environmental influences which vary across time and space, such as the weather [4]–[6]. A classic example of an ecological phenomenon caused by such environmental noise is the *Moran effect*, the tendency for a shared fluctuating environment to synchronize the variations in abundance among species and across space [7]–[10]. This effect has now been studied in systems with colored noise [11]–[13] and species dispersal [14], and in small food webs [15]–[19]. The synchronizing effect of noise, however, is opposed by negative interactions between species (e.g. through resource competition or predation) which cause *compensatory dynamics*: when the abundance of one species increases, the abundance of others tend to decrease, creating out-of-step variations [20]. Although significant progress has been made towards quantifying the total impact of each of these factors [21]–[23], it remains unknown how the tension between them influences the dynamics in natural ecosystems. In such systems, many phenotypically distinct species are embedded in a tangled web of direct and indirect interactions that make it hard to predict the effect of even simple disturbances [24]–[26], and non-trivial collective effects could play a significant role. For instance, even in the absence of noise species interactions can lead to static, clumped patterns across phenotype space [27], providing a possible explanation for the widely observed tendency for species in a given ecosystem to cluster around a few preferred body sizes [28], [29]. Such phenotypic patterns could be ubiquitous but have received relatively little attention [30].

The idea that the interplay between environmental noise and inter-species interactions could lead to non-trivial effects is supported by both theoretical and empirical studies of ecosystem dynamics. Even single- or few-species ecological models exhibit a range of complex behaviors, including bifurcations and chaos [31], strong amplification of environmental noise [32]–[34], noise-induced oscillations [35], [36], and pattern formation driven by demographic fluctuations [37]. Empirical observations in nature and laboratory experiments have similarly revealed complex dynamics, including chaotic behavior [38], [39], environmental noise and density-dependence intermingling in determining single species abundances [9], and cases where synchrony in the abundance of a single species across landscapes propagates down a food-web [40].

In this article, we show that environmental noise can indeed lead to robust, dynamic patterns in phenotype space. We introduce a simple model of the combined effect of noise and competition in an ecosystem with many species differing in their reliance on growth rate and efficiency, respectively, for survival. To focus on dynamically emerging patterns rather than on pre-imposed niche differences, we use a minimalist patch-model framework in which all species compete for a single resource and undergo periodic, global dispersal between the patches. Each species is entirely defined simply by its rate of growth and its efficiency in turning resources into offspring. We start by considering the model behavior in a fixed environment, showing that it allows many species to coexist stably. We then introduce external environmental noise and show that it gives rise to systematic and robust alternating patterns of species-species correlations which are accompanied by the formation of dynamic clusters of abundant species in phenotype space. Finally, we show that these patterns directly reflect a balance between the tendency of noise to synchronize different species and the tendency of competitive interactions to create abundance-differences.

## Results

### Ecosystems model

Our patch model is similar to both the theoretical model proposed by Wilson [41] and to (the metapopulation version of) the experimental yeast system of MacLean and Gudelj [42]. The specific formulation was inspired by the rich microbial communities found in soil (which exhibit many of the same broad ecological patterns as macroscopic species [43]), but its basic features – patchiness, repeated environmental disturbances, and the presence of a range of different phenotypic strategies – are shared by many ecosystems. In this sense, for instance, our model is similar to a model of competition between grasses analyzed by Tilman [44], [45]. Hence, we believe that our conclusions will also be relevant to many macroscopic ecosystems.

**Figure 1:**
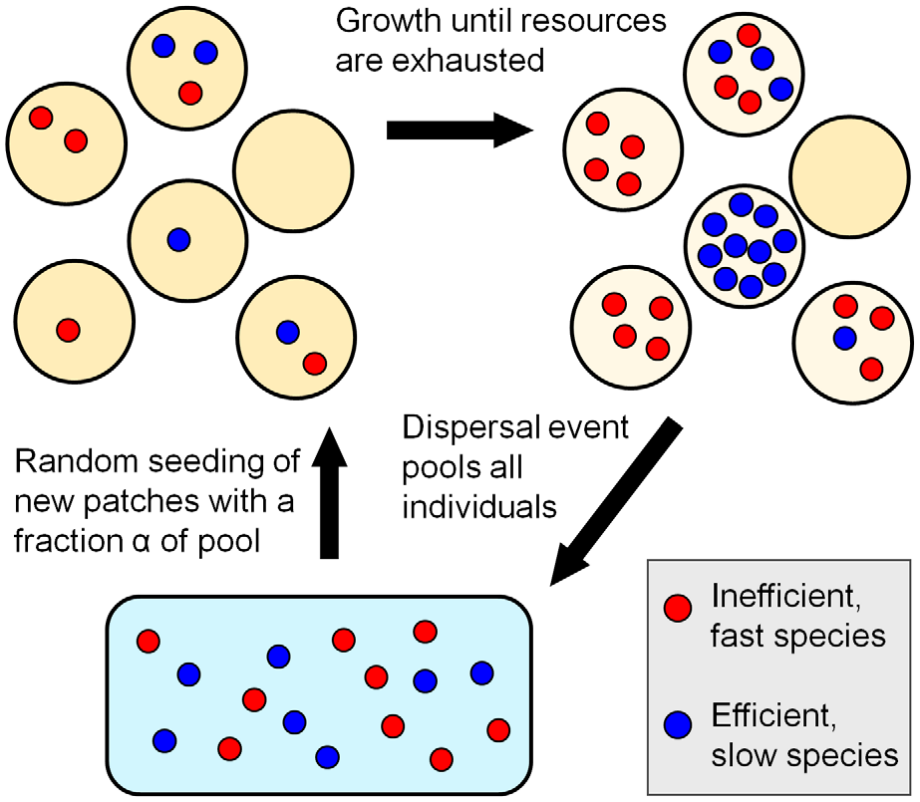
A grow-and-disperse patch model of competition for a single resource. Individuals (red, blue dots) are randomly distributed on identical nutrient patches (yellow discs) and grow exponentially until the single resource on their patch is exhausted. Once growth has ceased on all patches, a dispersal event collects all individuals into a seeding pool. The cycle then starts anew by seeding new patches with a fraction *α* of the individuals from the seeding pool. Each species is defined by two properties: its growth rate on a patch (*μ*) and its efficiency in turning resources into offspring (*Y*, number of offspring per patch in the absence of competitors) – for clarity, only two species are shown. An efficient but slow species (blue dots) grows to high densities when not subject to strong intra-patch competition, while the faster, inefficient species (red dots) has an advantage when competing with the slower species on the same patch. Since the seeding pool depends on the outcome of the previous round, a feedback loop regulates the selective pressure (see main text).

A key feature of the soil environment, as experienced by microbes, is its granular nature, with dividing cells typically found in separated pockets in the soil matrix [46]. These communities are not static: cells are constantly dispersed by weather and fresh resources are added and washed away continuously. Our model describes an ecosystem of *N* species competing for a single resource on multiple patches containing a fixed amount of the resource (Figure 1). The dynamics consists of repeated, two-phase cycles of local reproduction of individuals on their patches until the resource is depleted, followed by global dispersal to fresh patches (representing periodic environmental influence due to e.g. rainwater). The appearance of full nutrient patches can represent either the dispersal to existing but hitherto unoccupied locations or the addition of new resource by the environmental disturbance (e.g. deposited by water flow). Each species is described by two basic metabolic parameters, growth rate and efficiency [47], allowing us to consider the behavior of many species spread along continuous phenotype axes. Since efficiency would not confer an advantage unless resource availability is what limits growth, the model assumes that dispersal happens only after all resources have been exhausted. This assumption applies whenever disturbances are rare compared to the typical rates of growth, either because the dispersal events are intrinsically spaced out or because the resources are so finely divided that they only support short bursts of growth. An example of the first case is ecosystems where dispersal represents a yearly occurrence (e.g. for seeding plants), while the second case is likely to apply to e.g. microbes feeding off scattered organic matter in soil or the ocean (‘marine snow’ [48]).

For simplicity, we assumed that all nutrient patches are identical and always contain the same amount of resource at the beginning of a cycle. We also worked in the limit of infinitely many patches and hence infinitely large populations, allowing us to consider the impact of environmental noise on species abundance without complications due to demographic stochasticity.

Growth cycle number *t* starts with a global seeding pool in which the abundance per patch of each species is given by the vector **n**(*t*) = (*n_1_*(*t*), *n_2_*(*t*),…, *n_N_*(*t*)). From this pool, a fraction *α* of individuals randomly gets seeded onto a new collection of patches, while the remaining fraction, (1−*α*), of the cells is washed out of the system. We assumed *α* is very small so that the probability that a patch receives a total of *m_1_* individuals of species 1, *m_2_* of species 2 etc. is a product of Poisson probabilities:
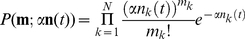
(1)where **m** = (*m_1_*, *m_2_*,…, *m_N_*). The two traits characterizing each species are: (1) growth rate, *μ* – the rate of exponential reproduction on a nutrient patch while resources are available, and (2) efficiency in turning nutrients into offspring, *Y* – the number of offspring that can be produced by a single individual if it consumes all the resource on a patch. After seeding, each individual of species *k* starts replicating at rate *μ_k_* while consuming the shared resource on its patch at a rate of 1/*Y_k_* units per offspring. Growth on a given patch stops when the resource on that patch is depleted. The time at which this happens (*T*) is a function of the initial abundance of each species on the patch, as well as of their growth rates and efficiencies, i.e. *T* = *T*(**m**;**μ**,**Y**), where the vectors **μ** and **Y** represent the growth and efficiency parameters for all species, respectively (see Methods). The final abundance of species *k*, averaged across all patches with this seeding, is then simply
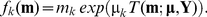
(2)


Since the interval between dispersal events is assumed to be longer than all growth-times, only the final abundances matter. The new average per-patch abundances, **n**(*t*+1), after all growth has stopped is found by averaging these final abundance over all possible seeding configurations:
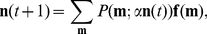
(3)where **f**(**m**) = (*f_1_*(**m**), *f_2_*(**m**),…, *f_N_*(**m**)). Equation 3 is the fundamental dynamical equation for the per-patch abundances at the end of growth phase. It expresses the fact that final species abundances in one cycle determine the abundances in the next by setting the probabilities of the various possible initial seedings. Details of the model and simulations are given in the Methods section.

We note that dispersal and the availability of new resources are assumed to be linked. Such linkage is natural if both are driven by the same external factor (e.g. rainfall dispersing bacterial cells and depositing new resources) or if one of them is driving the other. For instance, dispersal can effectively generate new resources if empty patches with new resources are always available and are simply being invaded by dispersal.

### Coexistence of many species

**Figure 2:**
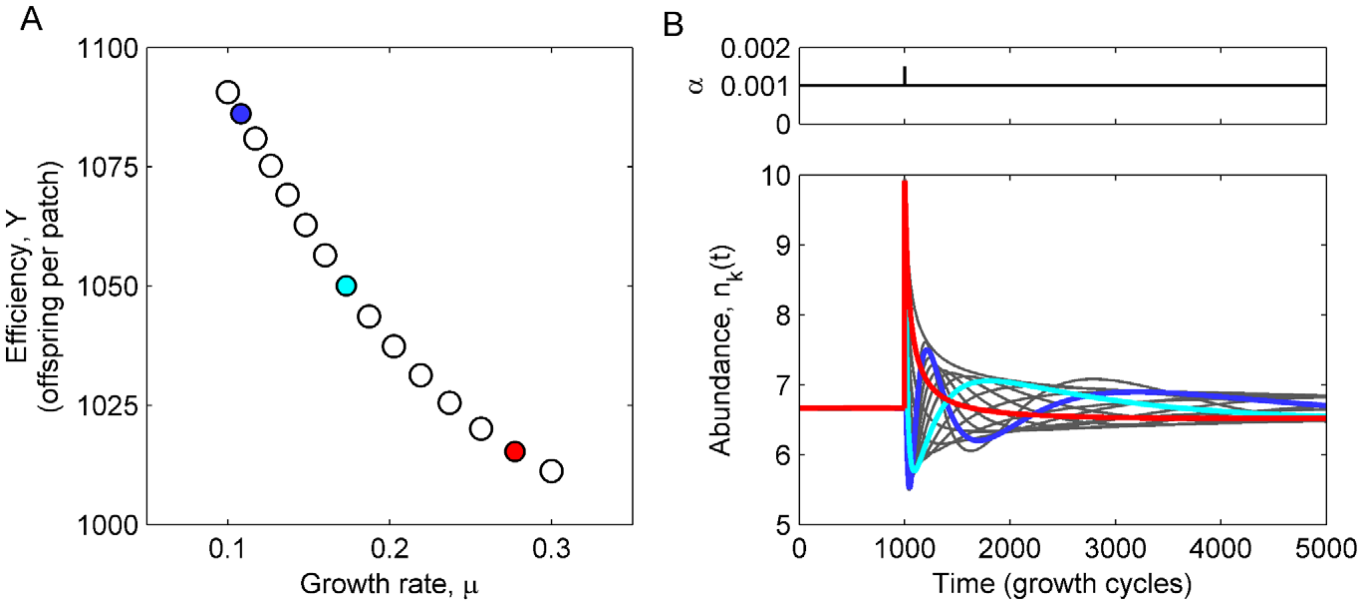
Multiple species can form stable communities given appropriate trade-off between growth rate and efficiency. (A) Growth rates and efficiencies for 15 species coexisting at fixed, equal densities (*n_k_* = 100/15 per patch; dispersal dilution *α* = 0.001). (B) Time-trace of the system in panel A started at the fixpoint, but subjected to a perturbation in *α* in cycle *t* = 1000 (spike in upper panel). The response of the 15 species is shown in the lower panel: the three representative species marked in color in panel A are shown with heavy lines, the remaining with thin grey lines. The perturbation drives the species abundances apart, but they relax back towards the steady state, indicating stable community coexistence (see also Methods).

While models of competition for a single resource typically lead to competitive exclusion – a single species comes to dominate and drives all others extinct [49], [50] – division into patches can allow many species to coexist [45], [51]. Indeed, numerical simulations of our model for fixed *α* showed that many species can be stably maintained (Figure 2), and it can be argued explicitly that arbitrarily many species can coexist if the amount of resource on each patch is very large (see Methods). The stabilizing mechanism that makes coexistence possible can be understood as a frequency-dependent selection during the growth-phase. When the total population density fluctuates up, patches are more likely to be seeded with more species, which intensifies competition and promotes selection for fast growth. If fast-growing species are also less efficient, their increased frequency drives the total population density back down. Conversely, when the population density is decreased, species have a higher probability of growing on patches with few or no competitors. This allows high-efficiency species to grow to high densities even if they are growing slowly, leading to an increase in the overall population. These growth-phase selection pressures – favoring speed (*μ*) and yield (*Y*), respectively– are examples of R- and K-selection [52], and can also be interpreted in terms of different levels of selection introduced by the division of the population into isolated groups [53].

The frequency-dependent fitness can lead to stable, steady-state solutions (fixpoints), n^*^, of Equation 3 such that **n**(*t+1*) = **n**(*t*) = n^*^: species abundances relax back to their steady state values following small perturbations (Figure 2). For such stabilization to work, however, constraints must prevent species from optimizing both growth and efficiency simultaneously and hence form a ‘super-species’ that will drive all other species extinct [50]. Cost-benefit reasoning suggests that such trade-offs will indeed generically be present, e.g. high efficiency will typically require more extensive metabolic machinery and hence divert energy away from cellular reproduction [54], and plants must divide their resources between e.g. root and seeds [55]. Such trade-offs have indeed been found empirically in a number of contexts [55]–[58], and trade-offs between the rate and efficiency of resource utilization has been shown to allow two distinct strains of yeast to coexist [42]. As our focus is on the dynamics of the ecosystem rather than its assembly through evolution, we will assume the existence of appropriate *μ*-*Y* trade-offs which allow community coexistence. Because of the stabilizing mechanism, trade-offs do not uniquely fix *μ* and *Y* for each species; instead, a range of different values are possible (each leading to different steady state abundances), albeit the range of parameters choices narrows as two species become very similar (Supplementary Figures S1 and S2). To have an unbiased baseline, we chose sets of parameters that lead to equal species abundance at steady state, i.e. *n_k_* ^*^ = *n_0_* for all species *k*. Given *n_0_*, **μ**, and *α*, we can numerically solve the fixpoint equation **n**(*t+1*) = **n**(*t*) for the species efficiencies **Y** using Equations 1 and 3 – see Figure 2A.

### Environmental noise leads to clustering of species in phenotype space

We introduced shared environmental noise through fluctuations in the dispersal dilution factor *α* which represents the strength of the environmental disturbance and affects all species in each step. Specifically, we drew an independent, random *α*-value in each cycle (white noise) from a fixed log-normal distribution. This choice is convenient for keeping the expectation value of the long-term dilution factor fixed as we changed the noise intensity, but our conclusions do not depends on the exact distribution (see Methods).

**Figure 3:**
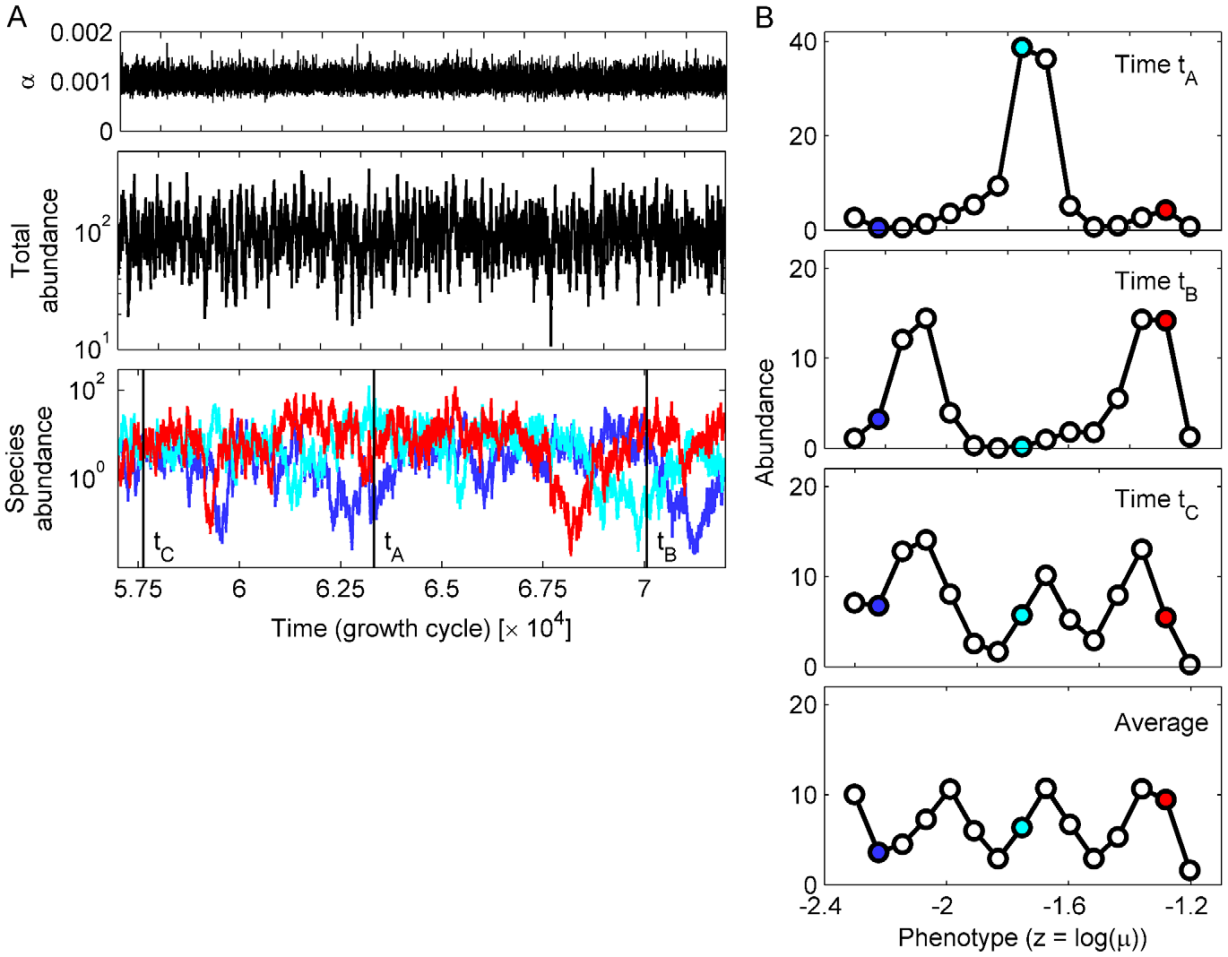
Environmental noise leads to clusters of abundant species in phenotype space. (A) Time-trace of the system from Figure 2 in the presence of noise in the dispersal dilution factor *α*. From top to bottom, the panels show: time-traces of *α*, the total per-patch abundance, and the per-patch abundances of the three representative species from Figure 2A. The dilution rate is drawn independently every cycle from a log-normal distribution with a coefficient of variation of *σ_α_*/<*α*> = 0.15. The total and individual abundances vary over several orders of magnitudes, with different species taking turns being the most abundant. (B) Examples of the species abundances profile in phenotype space at specific times (*t_A_*, *t_B_*, *t_C_*; indicated by vertical black lines in panel B), and the time-averaged profile. The species are arranged by increasing growth rate (the *x*-coordinates are *z_k_* = log(*μ_k_*)). The top three panels show cases of one, two, and three dominant peaks, examples of the typical peak-and-valley pattern induced by the environmental noise (see also Supplementary Figures S3 and S4). The number, position, and height of the peaks vary across time, but the uneven distribution remains when averaging over 50,000 time steps (bottom panel). Colored dots indicate the three representative species from panel A.

The environmental noise was strongly amplified: a 15% variation in *α* around the mean causes both the total abundance and that of individual species to fluctuate over several orders of magnitude (Figure 3A). Individual species exhibited short ‘bursts’ of high abundance and occasionally maintained a relatively high abundance over long periods. No single species permanently gained the upper hand – instead, there was a constant, slow turnover of species, reminiscent of that observed in plankton communities [59].

But while the fluctuations in the abundance of any single species are erratic, the competitive interactions acted to create a striking coherent pattern in the *relative* fluctuations of different species. At any typical time, the most abundant species formed clusters in phenotype space, separated by ‘valleys’ of low-abundance species (Figure 3B and Supplementary Figures S3, S4, and S5). Due to the turnover of dominant species, the number, and height of clusters changed over time, but the peak-and-valley pattern itself was robust. Furthermore, peaks tended to have approximately the same width in phenotype space. This clustered pattern remained when averaging over many cycles, albeit with a smaller amplitude (Figure 3B, bottom panel), and also appeared across replica systems started at different random configurations. Increasing the noise intensity has little impact on the typical size of the clusters, but naturally leads to larger abundance differences. At very high noise levels, non-linear effects – presumably related to the stabilizing mechanism discussed above – stabilizes rare species at low densities, leading to clusters separated by very distinct valleys (Supplementary Figure S6). Extinction of species can occur at very high noise levels, but was never observed at the noise strengths discussed in this paper.

### Environmental noise and multi-species interactions combine to create alternating correlations

**Figure 4:**
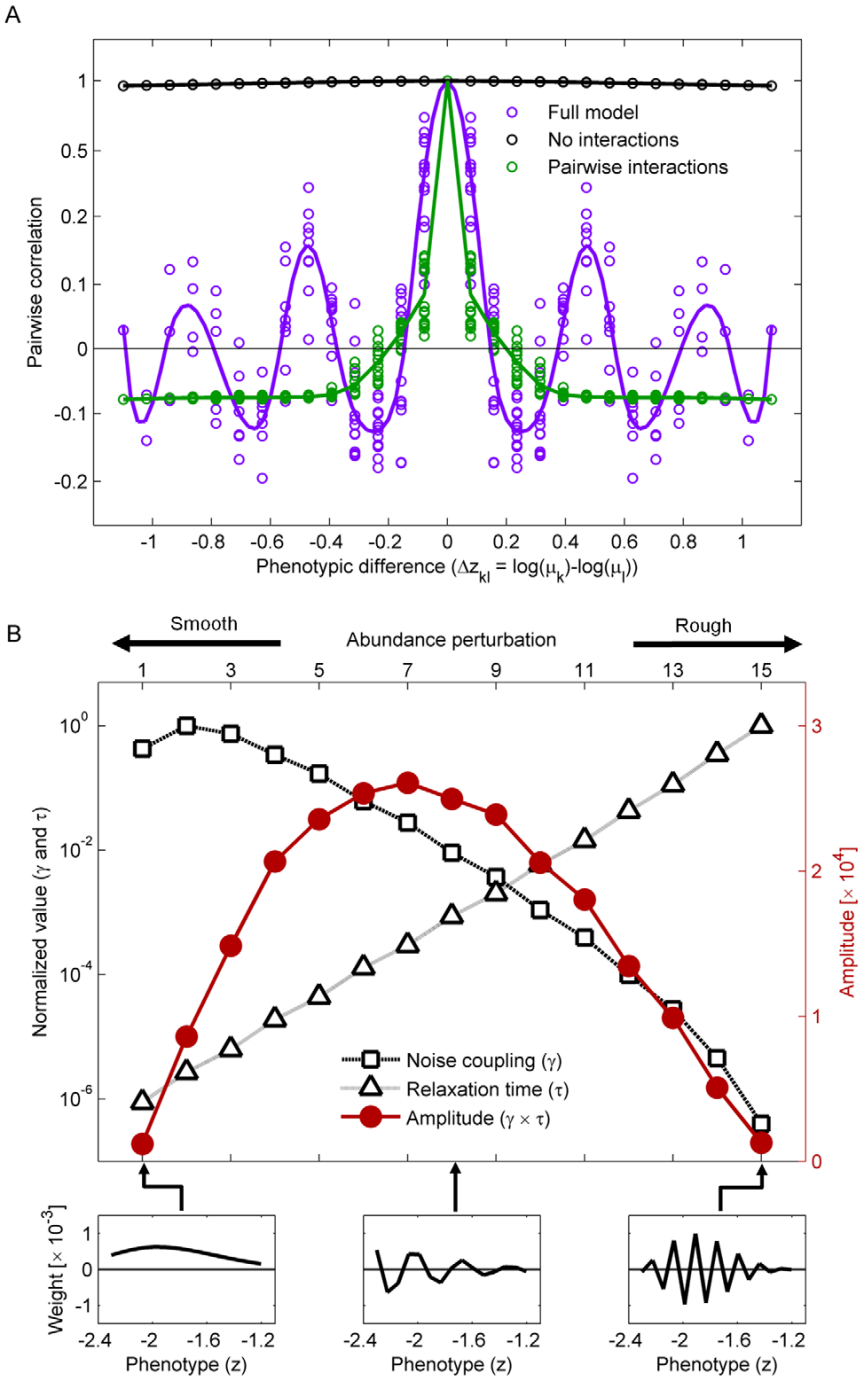
Alternating patterns of species correlation and clustering results from a balance between noise and many-species interactions. (A) Pair-wise correlations of species as a function of their phenotypic difference. Shown are the correlations in the full model (purple), and when only one species (No interactions, black) or two species (Pair-wise interactions, green) are allowed to fluctuate, all for the exact same noise-series in the dispersal dilution factor α (coefficient of variation 0.15). Dashed lines are splines through the mean values to guide the eye; the *y*-axis has been stretched near 0 to make details clearer. Without interaction, the individually fluctuating species show almost 100% correlations (Moran effect). Pair-wise interactions give rise to negative correlations between sufficiently different species (compensatory dynamics). However, only when all species are interacting do we see alternating correlations, indicating species clustering in phenotype space. Correlations are calculated from a data-series of 10^5^ steps. Since some timescales in the model are longer than simulations can feasibly be run, the correlations calculated depend on the length of the simulated run; however, here we are interested only in the contrast between the three patterns for a fixed simulation time. (B) Stability (Relaxation time, *τ*; triangles) and tendency to be generated by noise (Noise-coupling, γ; squares) for the 15 basic abundance perturbations around the steady state (eigenvectors of the linearized interactions, see Methods). The 15 perturbations are arranged by roughness, as illustrated by the three examples (bottom panels). In the presence of environmental noise, the contribution of each perturbation type is given by the amplitude curve (Amplitude, γτ; red circles). It peaks at medium smoothness where the typical abundance profile displays clustering (cf. the middle of the bottom panels). All quantities in panel B calculated from the linearized model; model parameters are as in Figures 2 and 3.

To understand how the phenotypic clusters are formed, we looked at the pair-wise correlation between species abundances in simulations of the complete model and constrained versions of it (data series of 10^5^ cycles). When plotted as a function of the phenotypic difference between them, the correlation between two species in the complete model alternates between positive and negative values (Figure 4A, purple), reflecting the clustering we observed in Figure 3B (since ‘peak-species’ move in synchrony with one another, but out of step with ‘valley-species’). To separate the contribution of noise and species interaction to this oscillatory pattern, we repeated the simulation with the exact same noise (same series of *α*-values) while artificially fixing the abundance of either all but one, or all but two species, to their steady state values. These two types of simulations maintain the properties of the steady state while singling out the contribution of the noise itself and the pair-wise interactions combined with noise, respectively. For the single-species version, we simulated each species separately (*N* simulation runs) and computed pair-wise correlations between the different simulations; for the pairs, we simulated all pairs (*N* ^2^ simulations) and computed the correlation of every pair of species within the corresponding simulation. We found that when each single species fluctuates independently, the full dynamics is determined by the noise and all species remain strongly positively correlated with each other regardless of how different they are (Figure 4A, black; no interactions – see also Supplementary Figure S5). Allowing pairs of species to fluctuate keeps similar species positively correlated, but causes species which are sufficiently phenotypically different become anti-correlated (Figure 4A, green; pair-wise interactions).

Hence, one- or two-species dynamics lead to the standard behaviors – Moran effect and compensatory dynamics, respectively. The latter effect is also visible in the response to an instantaneous increase in the abundance of a single species: the abundances of the other species drop (Supplementary Figure S7). The combination of noise and pair-wise interactions account correctly for the positive correlation between close species and for the negative correlation with some distant species, as seen in the complete model. However, pair-wise interactions alone are not sufficient for explaining the alternating patterns of multiple peaks of positive and negative correlations: this is a collective phenomenon requiring the interaction of many species. It only appears as we increase the number species allowed to fluctuate (Supplementary Figure S8).

### Clustering of species in phenotype space reflects a balance between the Moran effect and compensatory dynamics

The mechanism behind the species clustering in phenotype space can be understood as a dynamic balance between the smoothing (synchronizing) effect of noise and the roughening effects of interactions. When the system is perturbed by a change in the dilution parameter *α*, all the species change their abundances by similar amounts and in the same direction, generating a relatively smooth (uniform) change in the abundance profile across phenotype space. As shown above, if the species do not interact with each other they will move up and down in almost perfect lockstep and hence maintain a flat uniform profile (equal abundances). But if the species do in fact all compete, moving in lockstep means that every species experiences either increased or decreased competition from all the others after a perturbation and hence quickly gets pushed back to the fixpoint. If, for instance, all species simultaneously become more abundant, the resulting shortage of food will quickly decimate each one of them. Now suppose instead that the system is in a state where some species are above their fixpoint abundances and others below it – i.e. have an abundance profile that oscillates up and down. In that case, each species experiences a combination of *less* competition from species that are below their normal abundance and *more* competition from over-abundant species. These competitive differences partially cancel each other out, leading to a decreased pull on the abundance of each species and hence a slower relaxation back to the steady state. The more rugged the profile, the slower the relaxation: if similar species can have very different abundances, they can better cancel out each other's effects. We conclude that noise tends to generate smooth abundance profiles across phenotype space but, conversely, that the most stable profiles are the very jagged ones. We therefore expect that the typical abundance profile we observe is one that is neither completely flat nor maximally jagged, but instead changes smoothly between high and low abundances i.e. exhibits clusters of abundant species.

This heuristic argument can be tested rigorously by considering a simplified version of our model (Figure 4B). By expanding Equation 3 around the fixpoint n^*^ and keeping only the leading (linear) terms, we obtain a good approximation for weak noise (see Methods). The interactions between species are now described by a single *N×N* matrix *J*, and the eigenvectors of this matrix describe *N* independent deformations of the abundance profile around the steady state. These basic deformations can be sorted by their smoothness in phenotype space and are ordered accordingly on the *x*-axis in Figure 4B – three example profiles are illustrated in the bottom panels. The presence of both positive and negative elements in all but the first deformation is a direct reflection of compensatory dynamics: they involve some species growing more abundant while others become rarer. For each deformation, we calculated its propensity to be generated by noise (Figure 4B, squares), and the time it takes for it to decay back to the flat steady state (Figure 4B, triangles) – see Methods for details. The results confirm the argument above: the two properties change in opposite directions as the profiles become more jagged. The environmental noise tends to generate smooth deformations, but the jagged deformations are much more long-lived. Statistically, the typical profile will therefore be one showing smooth peaks a few species wide (Figure 4B, red line peaking at middle smoothness). Changing the noise intensity multiplies the amplitude of each deformation with the same constant and so does not affect the typical cluster size (see Methods). This analysis agrees excellently with what we observe in our simulations: persistent clustering, with clusters having the same typical size even though the exact abundance profile is constantly changing due to the stochastic noise (compare Figure 3C and the middle of the bottom panels in Figure 4B). The amplitude distribution (red line in Figure 4B) also agrees well with simulations (Supplementary Figure S9).

The linear analysis also reveals the origin of the strong noise amplification: Although the parameters were not chosen to bring this about, the system is very close to instability, with the most jagged abundance deformation taking *τ*∼10^7^ cycles to decay back to the fixpoint (for the parameters used in Figures 3 and 4). By the same token, a permanent shift in *α* (a press perturbation) will lead to significant shift in the stead-state abundances; the stabilizing mechanism discussed above acts only on changes in the abundances themselves (see also Supplementary Figure S10).

## Discussion

Our results show that the interplay between environmental noise and species interactions can induce robust patterns of alternating correlations between species abundances, leading to dynamic clustering of abundance in phenotype space. We demonstrated that the fundamental basis for this pattern is the dynamic balance between synchrony caused by noise (Moran effect) and the compensatory dynamics caused by the species interactions. Environmental noise is thus not merely a randomizing or synchronizing force, but can actively create ecological patterns that do not directly reflect fixed external factors like niches. These are collective phenomena requiring the presence of many species, suggesting that few-species ecological models may miss entire classes of dynamic behavior that could be important in natural ecosystems.

By pointing to environmental noise as an important structuring factor in ecosystems, these results could cast new light on a number of empirical observations. For instance, metabolic theory suggests that body mass *M* is linked to maximal growth rate through the scaling relation 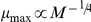 [60], so the clusters we observe across different growth rates could be directly reflected in cluster in the space of body mass. And indeed, body size cluster have been found to be dynamic in several cases, with the location of the clusters and their number changing over time [61]–[63]. Our model provides a simple mechanism for such itinerant clusters and at the same time offers a way to reconcile metabolic theory, which suggest the existence of single optimal body size, with the empirical observation that species rarely cluster at a single optimum [29]. Dynamic phenotypic clustering also implies that even species which are all direct competitors can arrange themselves into distinct sub-groups whose abundances fluctuate in synchrony for long periods of time (Figure 4A). This lends support to the suggestion that the apparent lack of strong negative correlations between species found in large-scale empirical studies [64]–[66] could be due to obscuring effects rather than the actual absence of negative interactions [67].

The formation of phenotypic clusters bears some resemblance to the classical concept of limiting similarity: the idea that competition puts a limit on how similar the phenotypes of coexisting species can be, and hence implying that two neighboring species must have a finite stretch of unoccupied phenotype space between them [68]. The sensitivity to environmental fluctuation in our model means that at a permanent shift in α could drive some species extinct and thus effectively lead to a new, larger phenotypic separation of neighboring species. Conversely, for Lotka-Volterra models it has been shown that a very small perturbation in the parameters can shift the system from allowing the coexistence of arbitrarily similar species to requiring a finite phenotypic difference [69]. If environmental fluctuations drive such an ecosystem back and forth between these two regimes fast enough to keep many species from going extinct, the result could be bands coexisting species similar to the clusters we observe.

As with all ecological modeling, we have made a number of simplifying assumptions. Firstly, we have ignored spatial structure beyond that provided by the division into patches. Secondly, we have worked in the limit of an infinite population size and hence neglected demographic noise (neutral ecological drift). Finally, we have assumed a pre-existing trade-off between efficiency and growth rate. The question of how such tradeoffs can evolve and how they affect ecosystem stability is complicated [70]–[73], and it would be interesting to understand it in the framework of our model. Indeed, the noise-induced clusters describe here could themselves play a role in speciation and the maintenance of genetic diversity [74]–[76].

Our model assumes that all patches contain the same amount of resource and deviations from this assumption are beyond the scope of this mode. However, we expect that if the resource amount on each patch was drawn independently from a fixed distribution in each round, the noise would simply average out and the model would converge to a steady state of coexistence similarly to that observed in our model. A slightly different natural variation would be to consider noise that affects the average amount of resources available on each patch rather than the dilution factor. A change in the amount of resource per patch is equivalent to a uniform rescaling of all efficiencies (see Methods) and therefore, like a change in dilution, will generically shift the balance between fast and slow species. We would therefore expect such fluctuations to cause qualitatively the same effects as we observe. Another possible variation of our model is to allow dispersal to occur before growth has finished on all patches. This would lower the advantage conferred by higher efficiency, so coexistence would require a steeper trade-off between growth-rate and efficiency. Indeed, in the limit of dispersal time much shorter than growth time, the model simply converges to exponential growth in a well- mixed environment; the efficiency becomes irrelevant and the fastest species takes over the population.

The appearance of dynamic phenotypic clusters in such a minimal simplified model suggest that species clustering in phenotype space could be a generic property of ecologies with many interacting species subject to noise. Indeed, the underlying mechanism is quite general and other noisy systems involving many interacting parts, e.g. neuronal or molecular networks, might exhibit similar effects. This mechanism could also work independently along several axes to create clusters in multi-dimensional phenotype spaces which could be seen as temporary ecological guilds [77]. Indeed, general metabolic theory suggests that body mass linked to many other ecological quantities by similar simple scaling relations [78] so if the clustering in the space of growth-rates transfer to body masses, as we argued above, it should also be reflected in patterns along still other phenotypic axes. It will be interesting to see whether such noise-induced abundance patterns can be directly observed in natural or laboratory-based experimental ecosystems, particularly microbial ones [79].

## Methods

### Model details and simulations

The full model is defined by Equations 1–3. To compute the final abundances for a given initial seeding, we first find the growth-time (*T*) given the available amount of resource, (*R*). Since all species grow freely, the number of offspring (not counting the original ancestor) of a single individual of species *k* at a time *t* is *exp*(*μ_k_t*)−1, and each new offspring removes 1/*Y_k_* units of resources. Starting from *m_k_* individuals, the total amount of resources consumed by the population of species *k* on a given patch is thus *m_k_*(*exp*(*μ_k_t*)−1)/*Y_k_*. Hence, *T* is the solution to the equation.
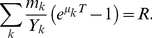
(4)


This equation defines a growth time *T* for every initial configuration **m**, given a set of growth rates **μ** and efficiencies **Y**. Changing the value of *R* is equivalent to scaling all the *Y*-values by a common factor, so we set *R* = 1 for convenience (this is the choice used in this paper). In that case, *Y* is simply the per-patch number of offspring produced by a single seeded individual in the absence of competitors.

We assumed that the environmental disturbances arrive at intervals longer than the time needed for even the slowest species to grow to saturation, i.e. the time between disturbances is longer than the largest *T*-value. Hence, the resources will always be completely exhausted on every patch and the time it took for this to happen (which varies depending on the seeding of the given patch) plays no further role. The final abundances for a given seeding *averaged over all patches with this seeding*, **f**(**m**), are now given by Equation 2. Using the average is consistent since we work with an infinite population; however, for a finite population, the stochastic growth differences between individual patches starting with the same seeding could change the results.

With the exception of the rather trivial case *N = 1*, we cannot analytically solve Equation 4, so we used numerical solutions for the simulations. Similarly, for *N*>1 we cannot analytically do the sum in Equation 3 since it depends on quantities than can only be found numerically. We therefore approximated it by summing over a finite number of seedings, imposing the condition that the combined probability of *all* neglected configurations was less than 10^−7^ (evaluated at the fixpoint). The resulting finite sum was over all seedings that involved at most *M* seeded individuals in total, where *M* was picked to satisfy the probability-condition. All simulations program were written in MATLAB and run on the Harvard Medical School supercomputing cluster (Orchestra).

### Coexistence of an arbitrary number of species

Because our model involves the solution of the transcendental Equation 4, a rigorous general proof of coexistence is difficult to provide. However, we can get close by drawing on similarities with the patch model of Tilman [45], in which simplified competitive dynamics makes it possible to prove that an arbitrarily large number of species can coexist.

Consider making the amount of resources on each patch very large or, equivalently, rescaling all efficiencies by a common large factor, *s*≫1:
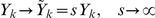



In this limit, the growth-time *T* clearly also goes to infinity. Expanding Equation 4, we see that on a given patch, *T* becomes dominated by the contribution from the highest-*μ* species present, with corrections due to other species falling off exponentially in *T*. Neglecting all but the fastest species and choosing an α such that 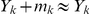 for all seedings that contribute significantly, we thus arrive at a ‘complete dominance approximation’ (for *R* = 1):
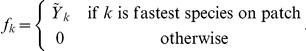



Plugging this into the dynamical equation, we can now do the sum and get a set of explicit fixpoint equations (we order the species so that *μ_1_*<*μ_2_*<…<*μ_N_*):
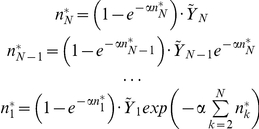



As in Tilman's model [45], the fixpoint abundance of a species now depends only on its own parameters and those of the species that are stronger competitors (have a higher *μ*). We can thus solve this hierarchy of equations for the efficiencies by working from the top and plugging the solution of each equation into those below. This allows us to find arbitrarily large sets of coexisting species.

### Structure of the environmental noise

We introduce environmental noise by drawing the dilution factor α from a log-normal distribution with probability density
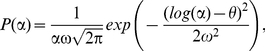
(5)where *θ* and *ω* are the mean and standard deviation of the *logarithm* of *α*, respectively. This gives a smooth, peaked distribution of tunable width that automatically implements the constraint that *α*>0. We made this choice since the long-term dilution rate – the expectation value of the product of many consecutive *α*s – is set by the expectation value of log(*α*) (cf. [80]) which we can control directly through *θ*. Had we instead kept the expectation value of *α* itself constant, we would have introduced changes in the expectation value of log(*α*) when changing the noise strength and hence biased the competition towards species that are either very efficient or very fast. To avoid this trivial bias, we kept *θ* constant as we increased the noise intensity (*ω*) in all simulations. Comparison with the linearized model (see below) shows that the exact choice of distribution for *α* is unimportant for the crucial features of the model.

### Fixpoint stability and the linear model

To test the stability of a fixpoint n*, we write **n**(*t*) = n*+Δ**n**(*t*) and *α*(*t*) = *α_0_*+Δ*α*(*t*), and expand the dynamic equation (Equation 3) in powers of Δ**n** and Δ*α* (*α_0_* is the dilution factor at the fixpoint). In the limit of low noise (Δ*α*/*α_0_*→0), the fluctuations will be small and we need only keep the leading terms. We thus arrive at the linear approximation:
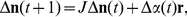
(6)where the matrix *J* and the vector **r** have elements
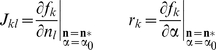
(7)(all derivatives evaluated at the fixpoint). The formulas for the derivatives can be derived from Equation 3, but must again be evaluated numerically for *N*>1. The fixpoint is stable if all eigenvalues *λ_k_* of the matrix *J* satisfy |*λ_k_*|<1 (complex modulus less than unity); we explicitly checked that this conditions was fulfilled this for the parameter sets used in the article. The dependence of the elements of *J* on the phenotypic distance between species is illustrated in panel (A) of Supplementary Figure S7.

We now introduce white, Gaussian noise defined by
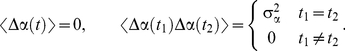
(8)where <…> indicate averages over the noise distribution. These are the only properties of the noise we will make use of, so the exact noise distribution will not play a role. We split the system into *N* independent eigenmodes by diagonalizing *J*:
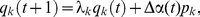
(9)where *λ_k_* is the *k* ^th^ eigenvalue of *J* (all real and positive for the parameters used), **p** = *S^−1^* **r**, and **q** = *S^−1^* **Δn** (the matrix *S* is the diagonalizing matrix whose columns are the *N* distinct eigenvectors of *J*). Using the noise properties (8) and the fact that |*λ_k_*|<1 (stable system), the average squared amplitude as *t→∞* is given by
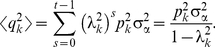
(10)


If we set *p_k_* = 0 (no noise), we find
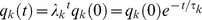
(11)where the relaxation time *τ_k_* is given by
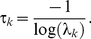
(12)


In our system, all the eigenvalues are real and close to 1, and can hence be written as *λ_k_ = 1−ε_k_* with 0<*ε_k_*≪1. Hence, we find
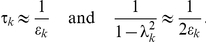



Therefore, we can write the equilibrium squared amplitude as a product of the coupling to the noise (*γ_k_*) and the relaxation time (*τ_k_*):
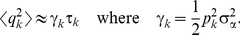
(13)


The values of *γ_k_*, (1−*λ_k_*)^−1^≈*τ_k_*, and 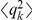 are plotted in Figure 4B (squares, triangles and red dots, respectively) – to facilitate visualization, the first two quantities have been rescaled so that their maximum value is 1. Notice that the noise strength *σ_α_^2^* appears as an overall factor and hence does not affect the shape of the amplitude spectrum.

The squared mode amplitudes for a simulated time-series of abundances, **n**(*t*), can be found simply by normalizing to the fixpoint and transforming into the eigenbasis:
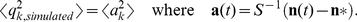
(14)


The average is performed over the simulated cycles. Comparisons of simulated data and the exact linear results from different number of species are shown in Supplementary Figure S9.

To each eigenvalue *λ_k_*, there corresponds an eigenvectors **v** *^(k)^* of *J*, the elements of which specifies a deformations of the abundances away from the fixpoint. For these deformations, the influence of each species is balanced so that they all return to the fixpoint at the same rate. Since the fast species are superior competitors, the components in each **v** *^(k)^* corresponding to fast species must therefore be correspondingly smaller. To make the oscillations in the profiles more visible, we have therefore plotted a weighted version of the profiles in Figure 4B. In the weighted eigenvectors 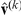, each component is multiplied by the average interaction the corresponding species has with other species, compensating for the trivial decrease in component values with competitive ability. The interaction between species in the linearized model is given by the matrix Δ*J* = *J−I*, where *I* is the unit matrix. The weighted eigenvectors thus have elements
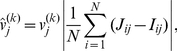
(16)where *I* is the unit matrix. The three plots below the main panel in ure 4B are plots of the components of the weighted vectors 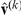 for *k* = 1, 8, and 15. For comparison, both the weighted and unweighted forms of these three vectors are plotted in Supplementary Figure S11.

## Supporting Information

Figure S1 Co-existence and relative abundances of two species across parameter space. The shaded area shows the range of parameters (*μ_B_*,*Y_B_*) that allows a second species B to coexist stably with a focal species A with (*μ_A_*,*Y_A_*) = (0.5,125) for *α* = 0.01 – the parameters of the focal species are marked with a ‘**+**’. The inset bar graphs show the fixpoint abundances of each species at three points in the coexistence region (marked with black dots). The fixpoint abundances vary from point to point: as we go from the lower edge of the shaded region to the top/right edge, we go from A being dominant to equal abundances and, finally, to B dominating. We can thus vary the relative abundances without destroying coexistence.
(TIF)

Figure S2 Constraints on the choice of parameters for similar species varies with α. The plot shows the range of efficiencies allowed for a given species when requiring it to coexists with a single other, similar species with parameters (*μ_A_ Y_A_*) = (0.5, 1200). Plotted is the width of the *Y*-interval over which coexistence is possible (*Y_B_^max^*−*Y_B_^min^*) for a given *μ_B_*, as a function of *α*. Each curve corresponds to a different μ_B_, all slightly larger than *μ_A_*. The allowed ranges narrow with increasing *α*.
(TIF)

Figure S3 Clustering is generic I. Parameters identical to the full model plots in Figure 3, except that the system was started with a different set of random abundances.
(TIF)

Figure S4 Clustering is generic II. Identical to the full model plots in Figure S3, except that the system was started with yet a different set of random abundances.
(TIF)

Figure S5 Clustering depends on interactions. Examples of instantaneous abundance distributions at 12 randomly selected time-points out of a 100,000-cycle time-series, with competition (filled circles) and without competition (unfilled circles) between species (the cycle no. is given above each plot). Without interactions, the distribution remains flat, but with competition it generically shows one or more clusters. The full-model data series is the same as the one used in Figure 3. The data without interactions is the single-species data series used in Figure 4A.
(TIF)

Figure S6 Cluster amplitude, but not their typical size, changes with noise intensity. The rows shows examples of abundance-snapshots of typical clusters for separate simulation with noise intensities (A) σ_α_/<α> = 0.045, (B) σ_α_/<α> = 0.15, and (C) σ_α_/<α> = 0.36 – notice the different scales on the vertical axes. Increasing the noise intensity leads to larger abundance differences and, for very high levels, clusters separated by distinct valleys of rare species. Model parameters as in Figures 3 and 4.
(TIF)

Figure S7 Response of ecosystem to abundance perturbations shows compensatory dynamics. (A) The linearized response to a perturbation in the abundance of a single species, as given by the elements of the matrix *J* (see Eqn. 7). The elements *J_kl_* are shown for three representative perturbed species (*k* = 2, 8, 14). All values are negative, indicating compensatory dynamics. The response of the species being perturbed is not shown. (B) Response of the full system (in steady-state) to a sudden increase in the abundance of a single species, as shown by the deviation of the abundances from their fixpoint values 10 steps after the perturbation. Examples species as in panel A, perturbed species not shown. Again, we find compensatory dynamics. System parameters as in Figure 4A.
(TIF)

Figure S8 Change in correlation structure with increasing number of interactions. (A) Pairwise correlations between species as a function of their phenotypic difference for a system in which 10 species are kept at their fixpoint abundances while the rest are allowed to fluctuate. Data based on 25 replica simulation in which the species allowed to fluctuate were randomly selected and the correlations between every pair of species within each simulation calculated. Grey points are individual results, black line is a spline fit to the mean value for each phenotype difference. (B) As panel A, but with only 5 species fixed. We see that as we increase the number of species that fluctuate, the correlation shifts from a mostly flat, purely compensatory pattern to the oscillatory pattern characteristic of the full model (compare with the full model and pairwise interaction curves in Figure 4A). System and runtime parameters as in Figure 4A; fluctuating species started at random abundances.
(TIF)

Figure S9 Comparison of linear analytical results and simulations. The amplitude of the various deformations for systems with 6, 10, and 15 species – curves show the exact result for the linearized model (red), and for the full model simulated at low (*σ_α_*/<*α*> = 0.045, blue) and higher noise (*σ_α_*/<*α*> = 0.15, black). As in Figure 4B in the main text, the perturbations are ordered by their by their roughness. The finite simulation time (10^5^ cycles in all cases) implies that the slowest deformation cannot be fully captured in simulations (all perturbations to the right of the vertical, dotted line have relaxation times longer than the simulated time-span). With only 6 species, the longest relaxation time is only ∼3,000 cycles. Hence, the 10^5^-step simulation captures the full behavior of all deformations and shows excellent agreement with the linear approximation. With 10 species, the longest time-scale is above 10^5^ cycles and greater deviations are seen at the slowly-relaxing deformations. This effect is even more marked for 15 species, but the agreement is still good for the smoother deformations. Specifically, the crucial feature – amplitude peaking at medium-smooth deformations – remains. The 15-species system is identical to that used in all figures in the main text. For all three cases, the species are logarithmically spaced between *μ* = 0.1 and *μ* = 0.3, and the fixpoint dilution factor is *α* = 0.001. The curves for 6 species are based on a single simulation; the ones for 10 and 15 species are averages of 5 simulations with different noise-series and starting abundances.
(TIF)

Figure S10 The response of a fixpoint community to a perturbation in α. The plot shows the derivative *dn_k_/dα* (evaluated at the fixpoint) for all species *k*. The system responds very sensitively to changes in *α* (*dn_k_/dα*∼6000), but the response shows little variation between species. Parameters as in Figures 3 and 4.
(TIF)

Figure S11Weighted and raw forms of the basic abundance perturbations. The upper row shows the weighted vectors 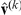 for *k* = 1,8,15. The bottom row shows the corresponding unweighted eigenvectors **v** *^(k)^*.(TIF)
