# Supplementary figures and images for: Dynamic Phenotypic Clustering in Noisy Ecosystems
Source: PLoS Comput Biol. 2011 Mar 17;7(3):e1002017. doi: 10.1371/journal.pcbi.1002017 (PMC3060162; doi:10.1371/journal.pcbi.1002017)

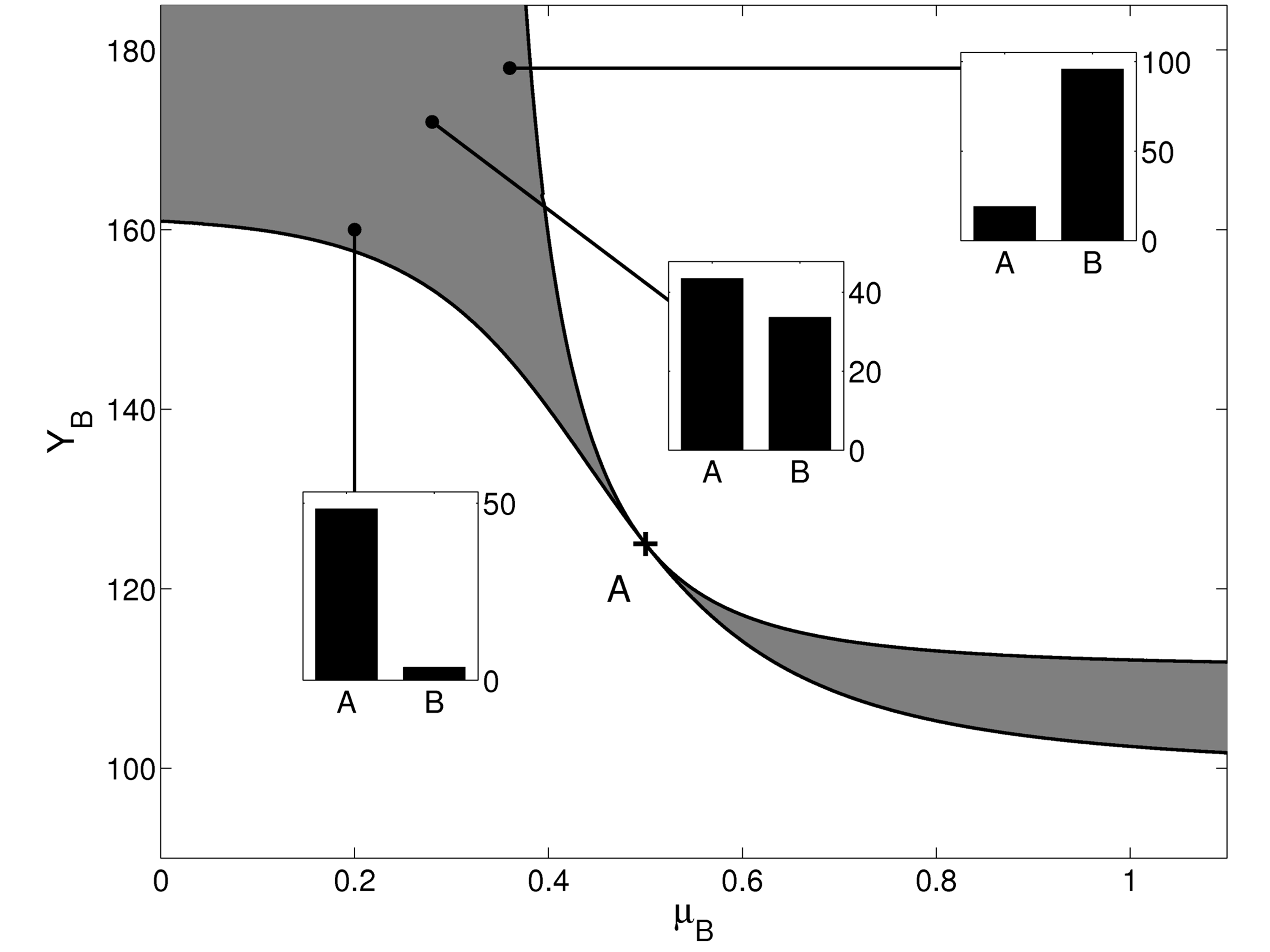

Supplement: Figure S1 — Co-existence and relative abundances of two species across parameter space. The shaded area shows the range of parameters (μB,YB) that allows a second species B to coexist stably with a focal species A with (μA,YA) = (0.5,125) for α = 0.01 – the parameters of the focal species are marked with a ‘+’. The inset bar graphs show the fixpoint abundances of each species at three points in the coexistence region (marked with black dots). The fixpoint abundances vary from point to point: as we go from the lower edge of the shaded region to the top/right edge, we go from A being dominant to equal abundances and, finally, to B dominating. We can thus vary the relative abundances without destroying coexistence. (TIF) [file pcbi.1002017.s001.tif]

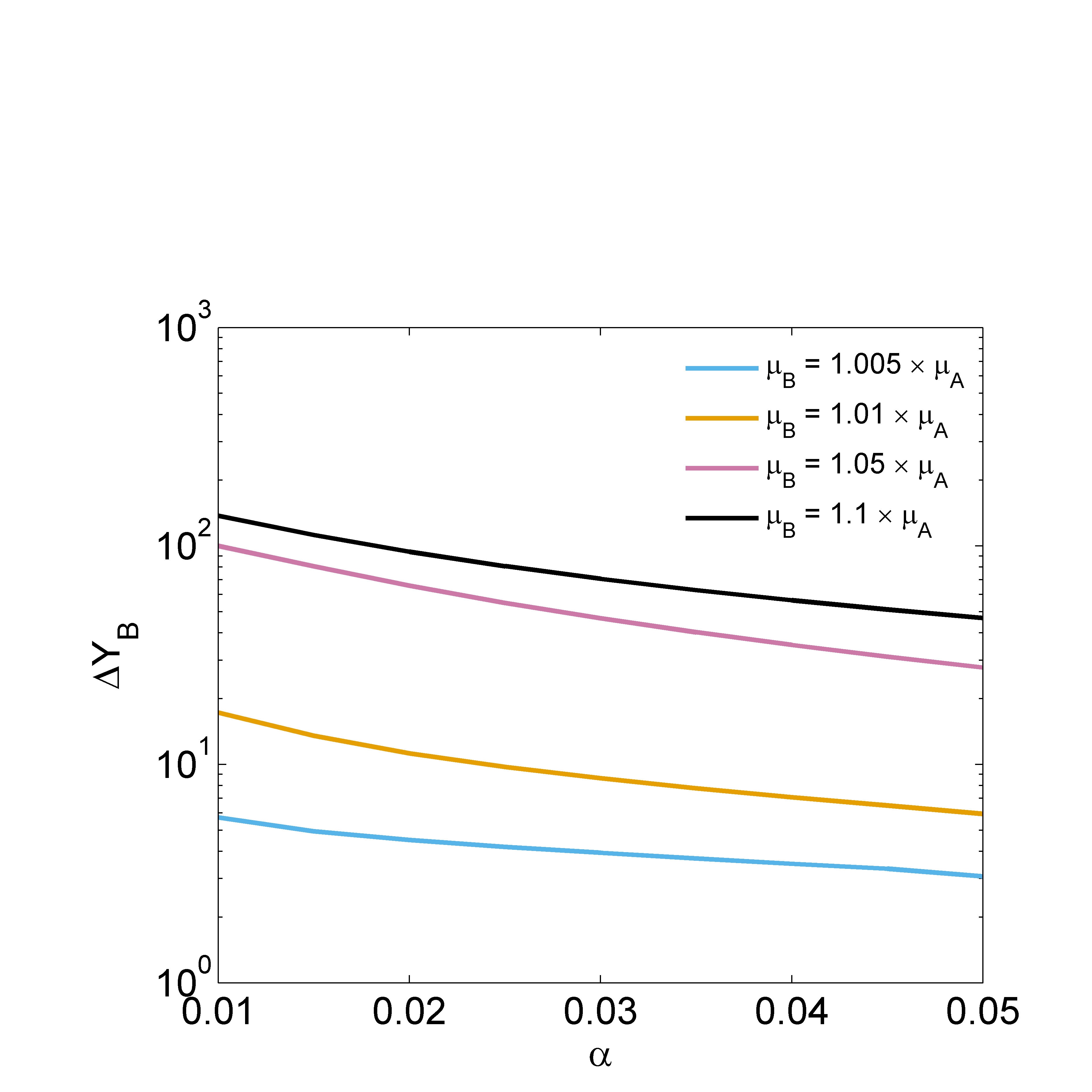

Supplement: Figure S2 — Constraints on the choice of parameters for similar species varies with α. The plot shows the range of efficiencies allowed for a given species when requiring it to coexists with a single other, similar species with parameters (μA YA) = (0.5, 1200). Plotted is the width of the Y-interval over which coexistence is possible (YBmax−YBmin) for a given μB, as a function of α. Each curve corresponds to a different μB, all slightly larger than μA. The allowed ranges narrow with increasing α. (TIF) [file pcbi.1002017.s002.tif]

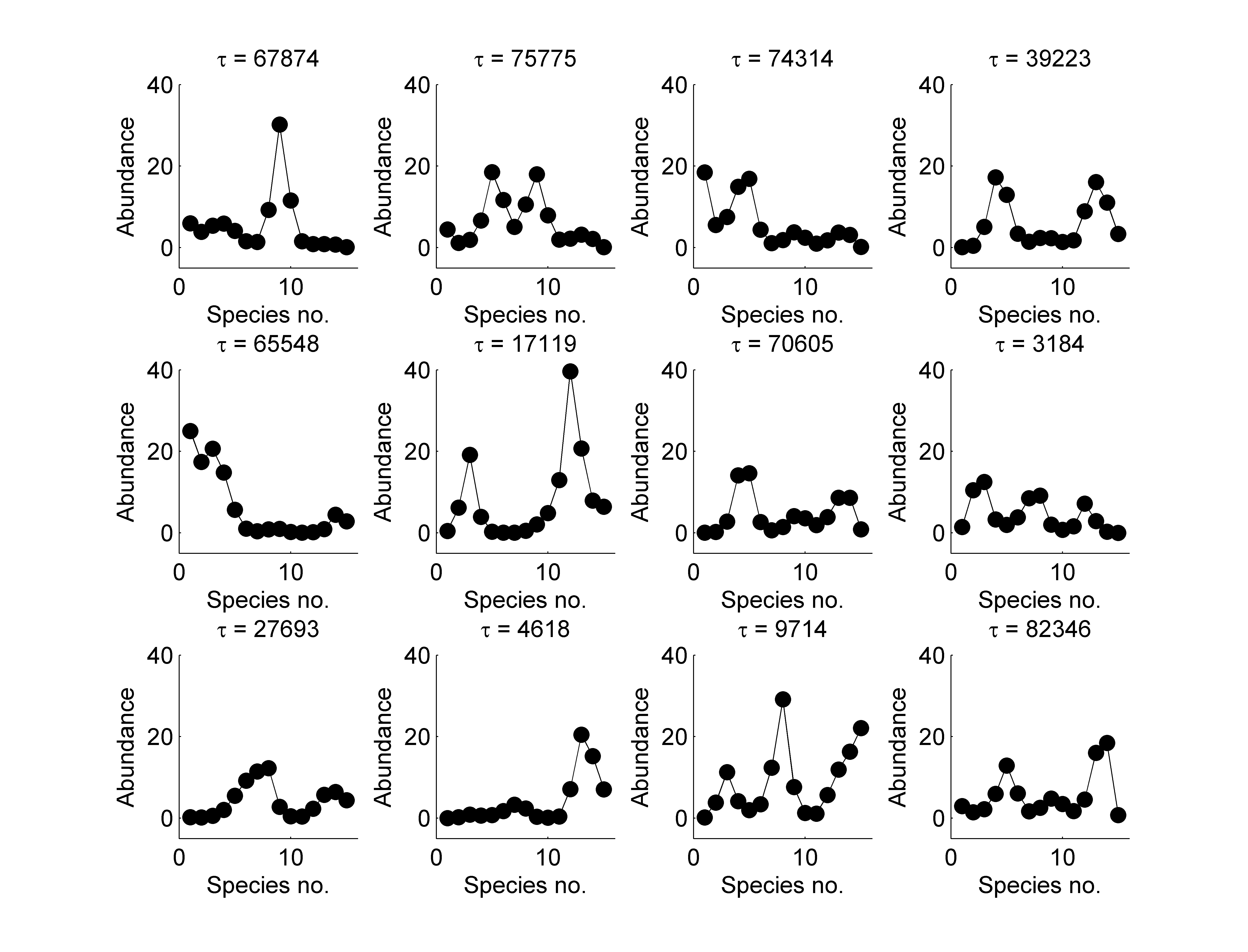

Supplement: Figure S3 — Clustering is generic I. Parameters identical to the full model plots in Figure 3, except that the system was started with a different set of random abundances. (TIF) [file pcbi.1002017.s003.tif]

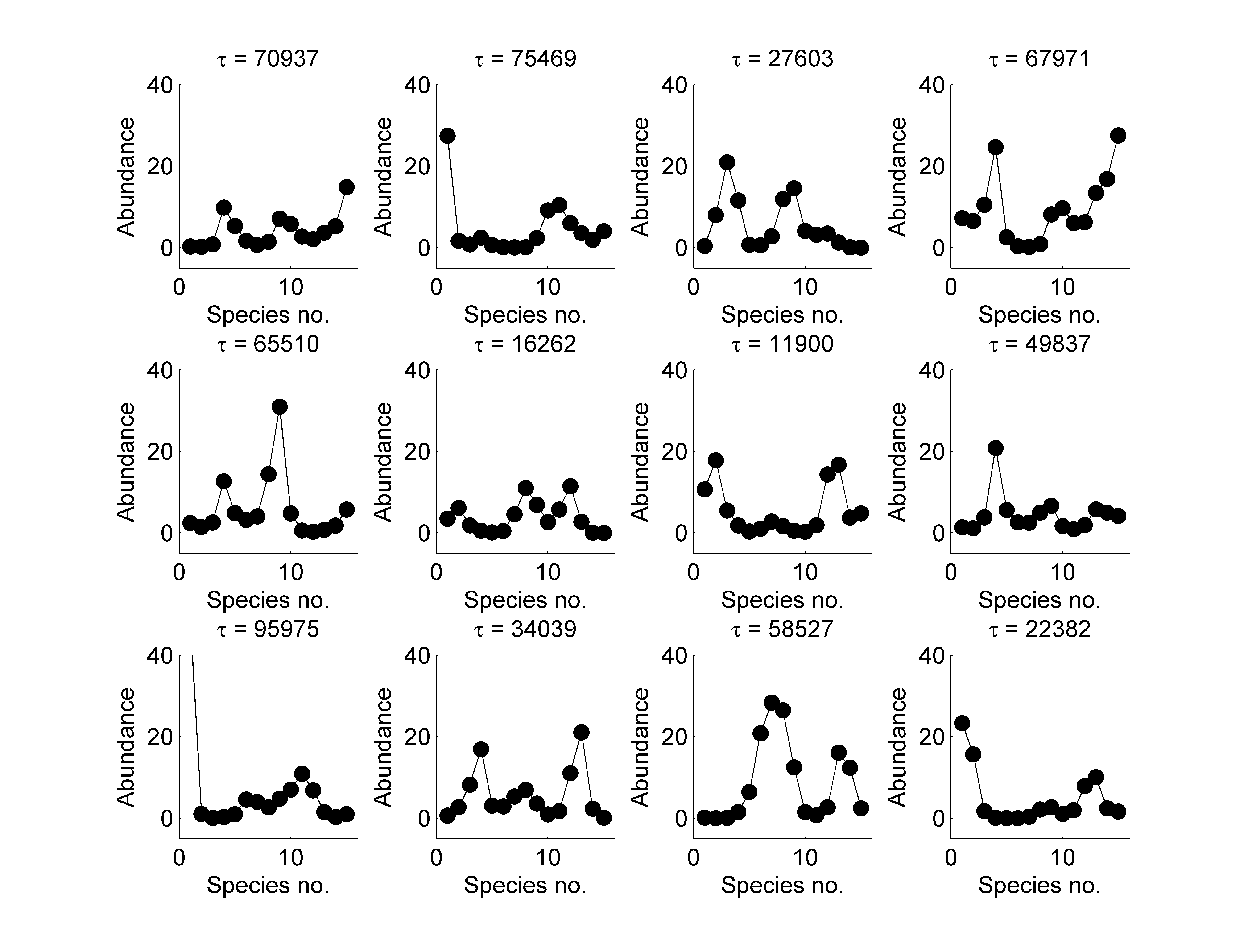

Supplement: Figure S4 — Clustering is generic II. Identical to the full model plots in Figure S3, except that the system was started with yet a different set of random abundances. (TIF) [file pcbi.1002017.s004.tif]

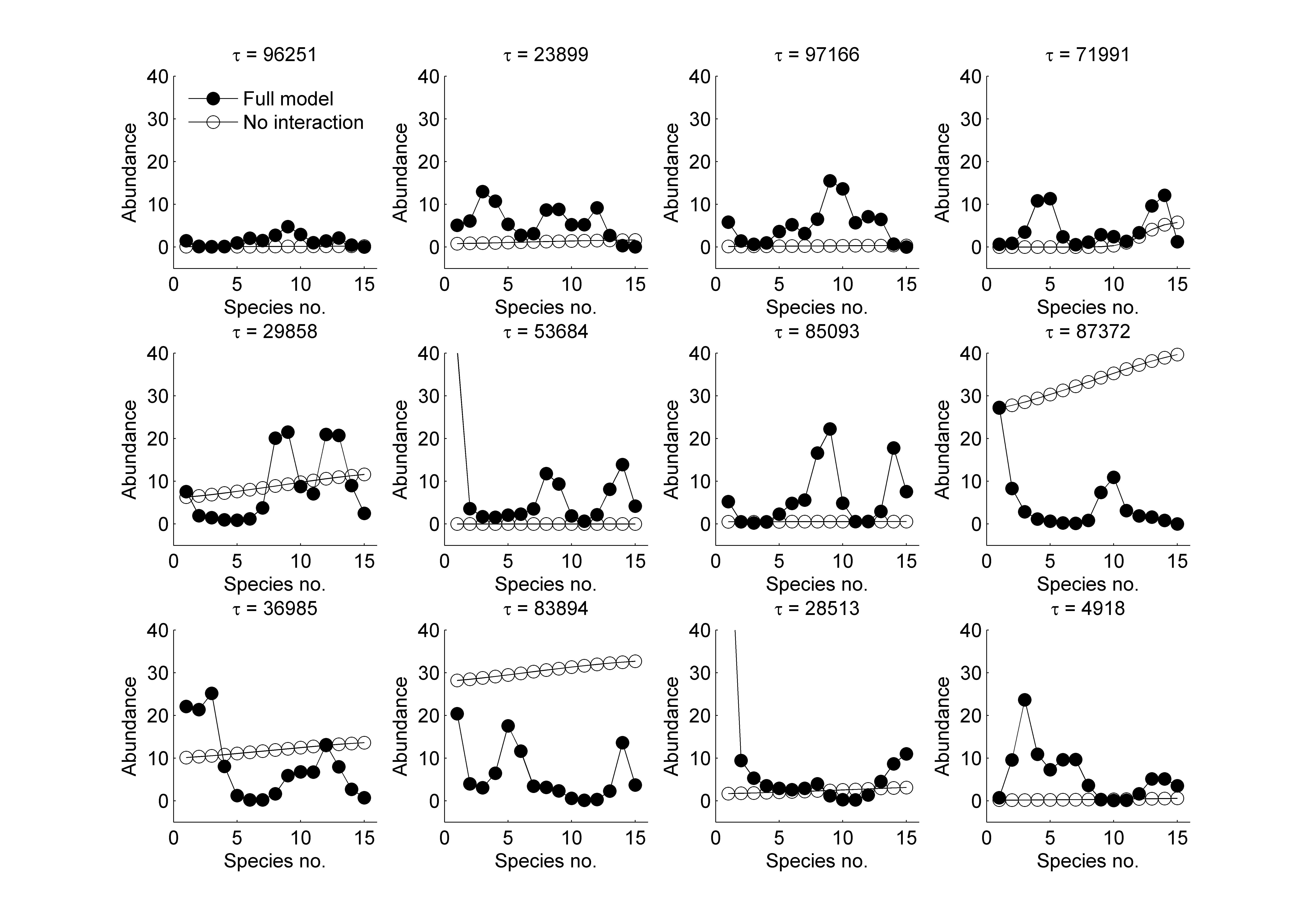

Supplement: Figure S5 — Clustering depends on interactions. Examples of instantaneous abundance distributions at 12 randomly selected time-points out of a 100,000-cycle time-series, with competition (filled circles) and without competition (unfilled circles) between species (the cycle no. is given above each plot). Without interactions, the distribution remains flat, but with competition it generically shows one or more clusters. The full-model data series is the same as the one used in Figure 3. The data without interactions is the single-species data series used in Figure 4A. (TIF) [file pcbi.1002017.s005.tif]

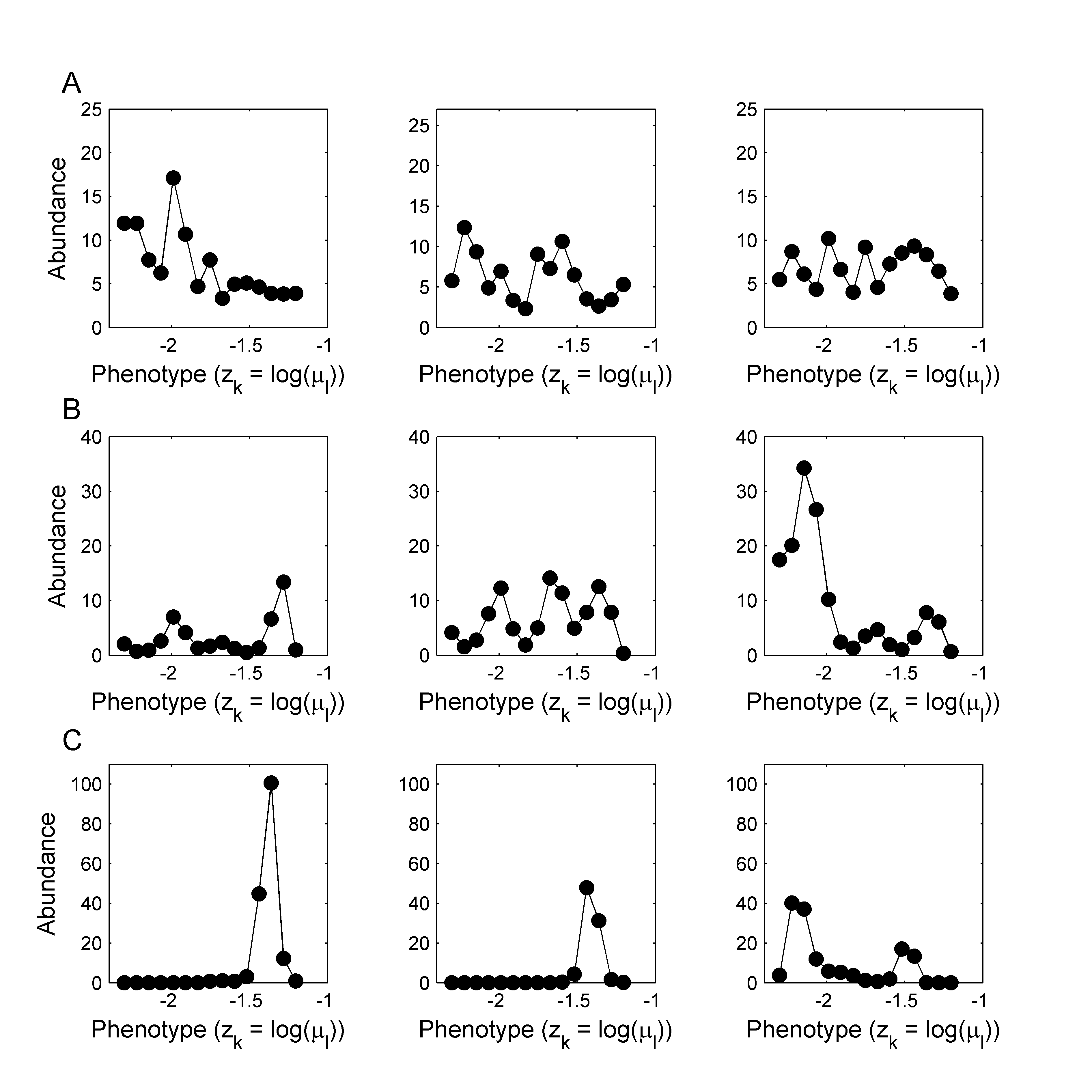

Supplement: Figure S6 — Cluster amplitude, but not their typical size, changes with noise intensity. The rows shows examples of abundance-snapshots of typical clusters for separate simulation with noise intensities (A) σα/<α> = 0.045, (B) σα/<α> = 0.15, and (C) σα/<α> = 0.36 – notice the different scales on the vertical axes. Increasing the noise intensity leads to larger abundance differences and, for very high levels, clusters separated by distinct valleys of rare species. Model parameters as in Figures 3 and 4. (TIF) [file pcbi.1002017.s006.tif]

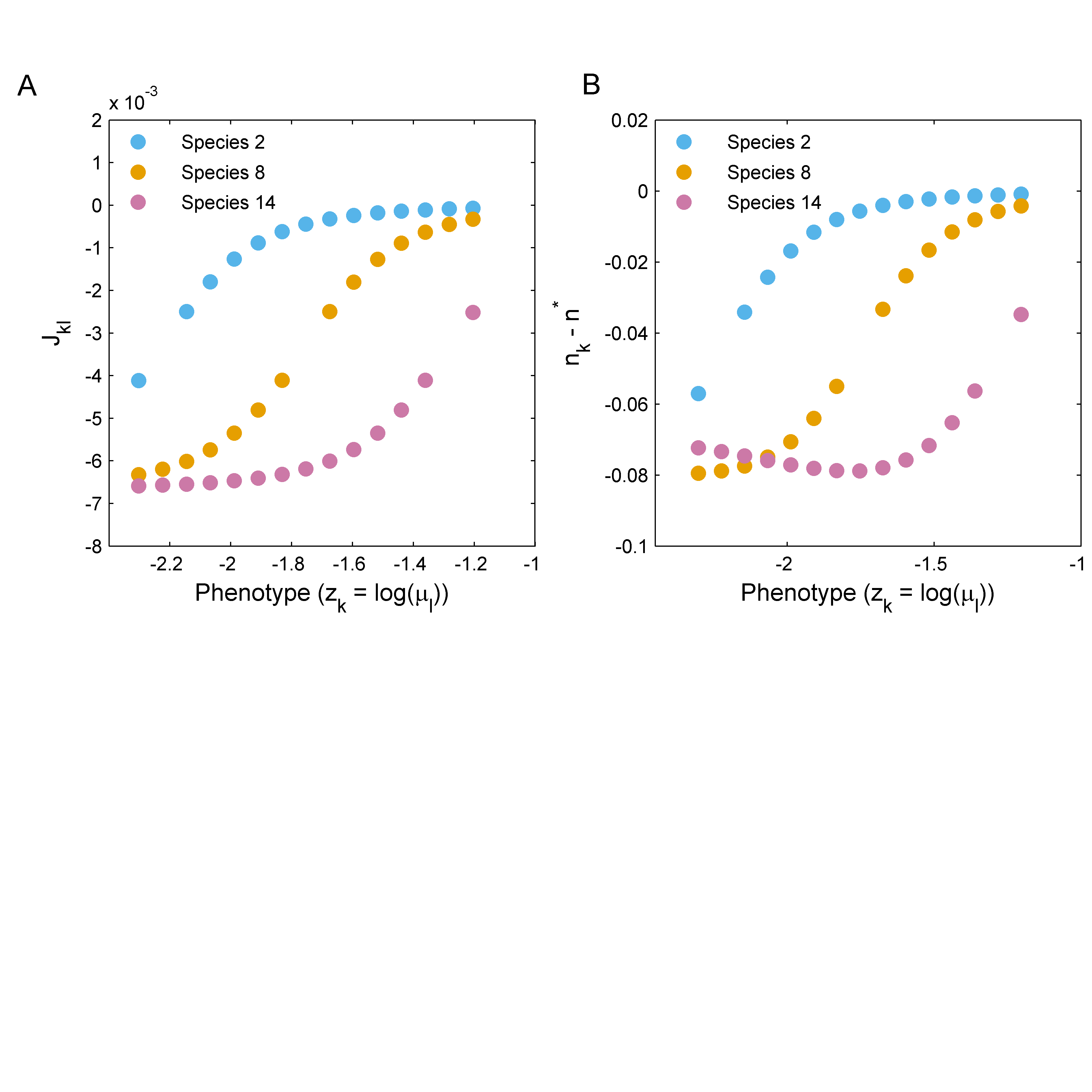

Supplement: Figure S7 — Response of ecosystem to abundance perturbations shows compensatory dynamics. (A) The linearized response to a perturbation in the abundance of a single species, as given by the elements of the matrix J (see Eqn. 7). The elements Jkl are shown for three representative perturbed species (k = 2, 8, 14). All values are negative, indicating compensatory dynamics. The response of the species being perturbed is not shown. (B) Response of the full system (in steady-state) to a sudden increase in the abundance of a single species, as shown by the deviation of the abundances from their fixpoint values 10 steps after the perturbation. Examples species as in panel A, perturbed species not shown. Again, we find compensatory dynamics. System parameters as in Figure 4A. (TIF) [file pcbi.1002017.s007.tif]

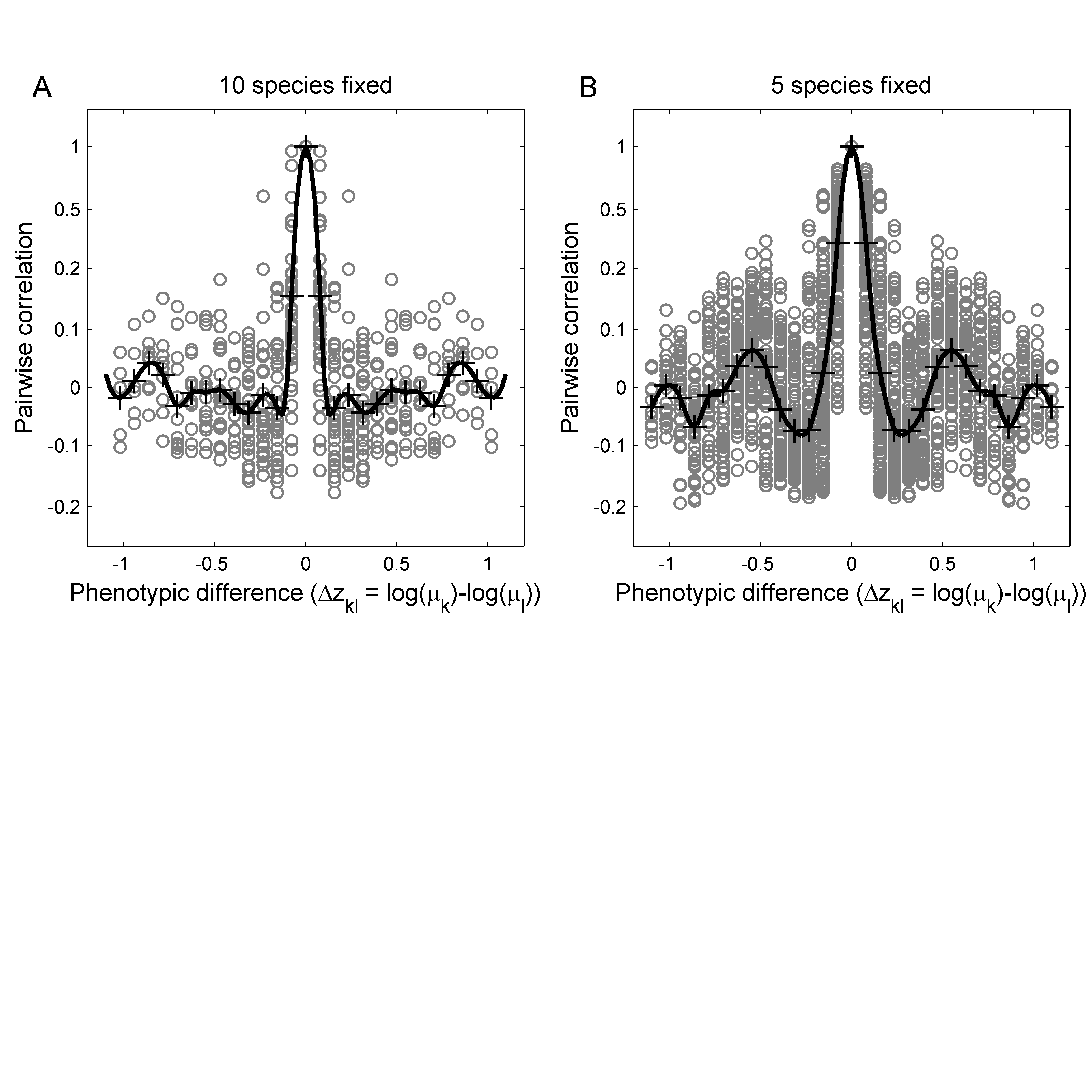

Supplement: Figure S8 — Change in correlation structure with increasing number of interactions. (A) Pairwise correlations between species as a function of their phenotypic difference for a system in which 10 species are kept at their fixpoint abundances while the rest are allowed to fluctuate. Data based on 25 replica simulation in which the species allowed to fluctuate were randomly selected and the correlations between every pair of species within each simulation calculated. Grey points are individual results, black line is a spline fit to the mean value for each phenotype difference. (B) As panel A, but with only 5 species fixed. We see that as we increase the number of species that fluctuate, the correlation shifts from a mostly flat, purely compensatory pattern to the oscillatory pattern characteristic of the full model (compare with the full model and pairwise interaction curves in Figure 4A). System and runtime parameters as in Figure 4A; fluctuating species started at random abundances. (TIF) [file pcbi.1002017.s008.tif]

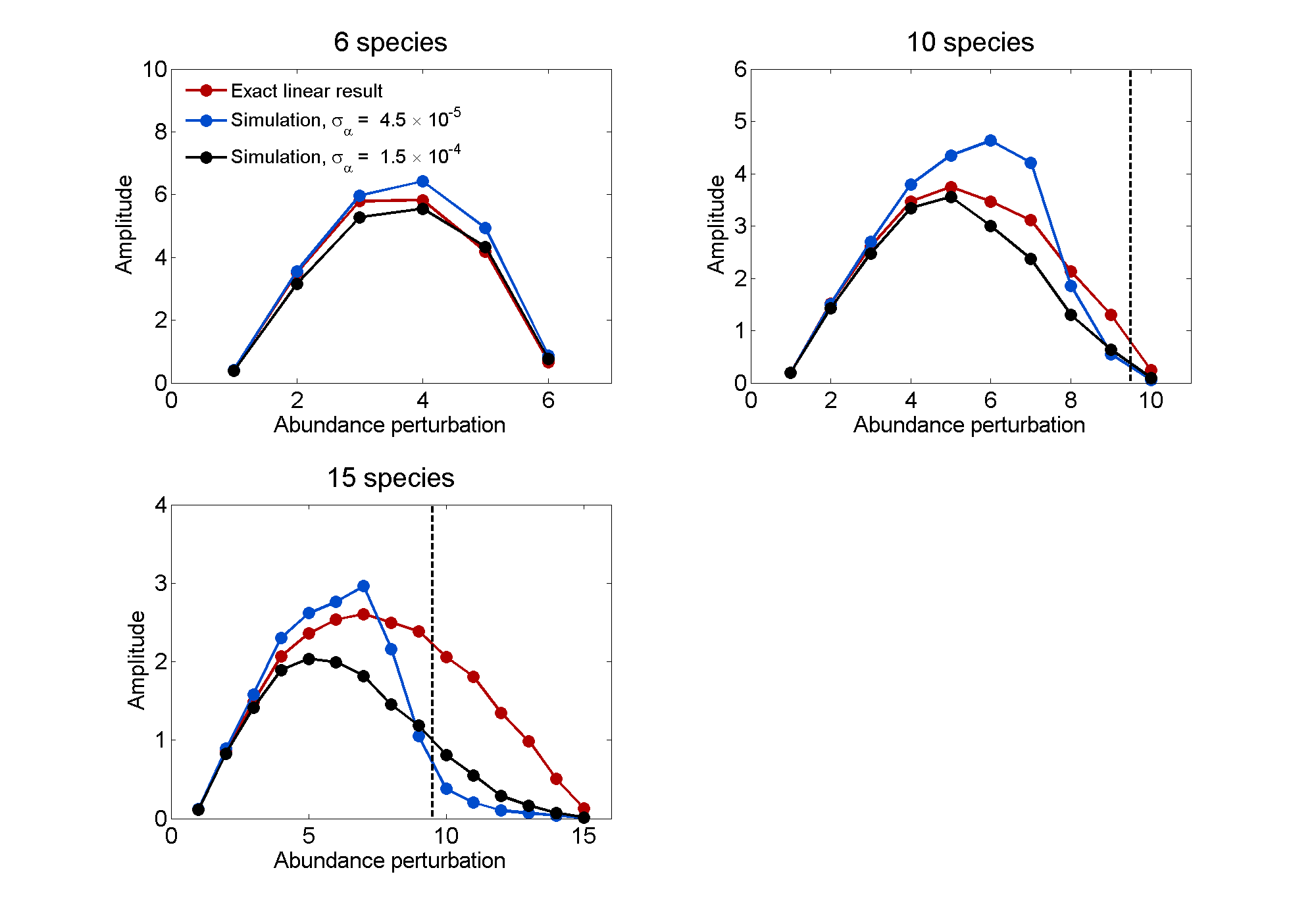

Supplement: Figure S9 — Comparison of linear analytical results and simulations. The amplitude of the various deformations for systems with 6, 10, and 15 species – curves show the exact result for the linearized model (red), and for the full model simulated at low (σα/<α> = 0.045, blue) and higher noise (σα/<α> = 0.15, black). As in Figure 4B in the main text, the perturbations are ordered by their by their roughness. The finite simulation time (105 cycles in all cases) implies that the slowest deformation cannot be fully captured in simulations (all perturbations to the right of the vertical, dotted line have relaxation times longer than the simulated time-span). With only 6 species, the longest relaxation time is only ∼3,000 cycles. Hence, the 105-step simulation captures the full behavior of all deformations and shows excellent agreement with the linear approximation. With 10 species, the longest time-scale is above 105 cycles and greater deviations are seen at the slowly-relaxing deformations. This effect is even more marked for 15 species, but the agreement is still good for the smoother deformations. Specifically, the crucial feature – amplitude peaking at medium-smooth deformations – remains. The 15-species system is identical to that used in all figures in the main text. For all three cases, the species are logarithmically spaced between μ = 0.1 and μ = 0.3, and the fixpoint dilution factor is α = 0.001. The curves for 6 species are based on a single simulation; the ones for 10 and 15 species are averages of 5 simulations with different noise-series and starting abundances. (TIF) [file pcbi.1002017.s009.tif]

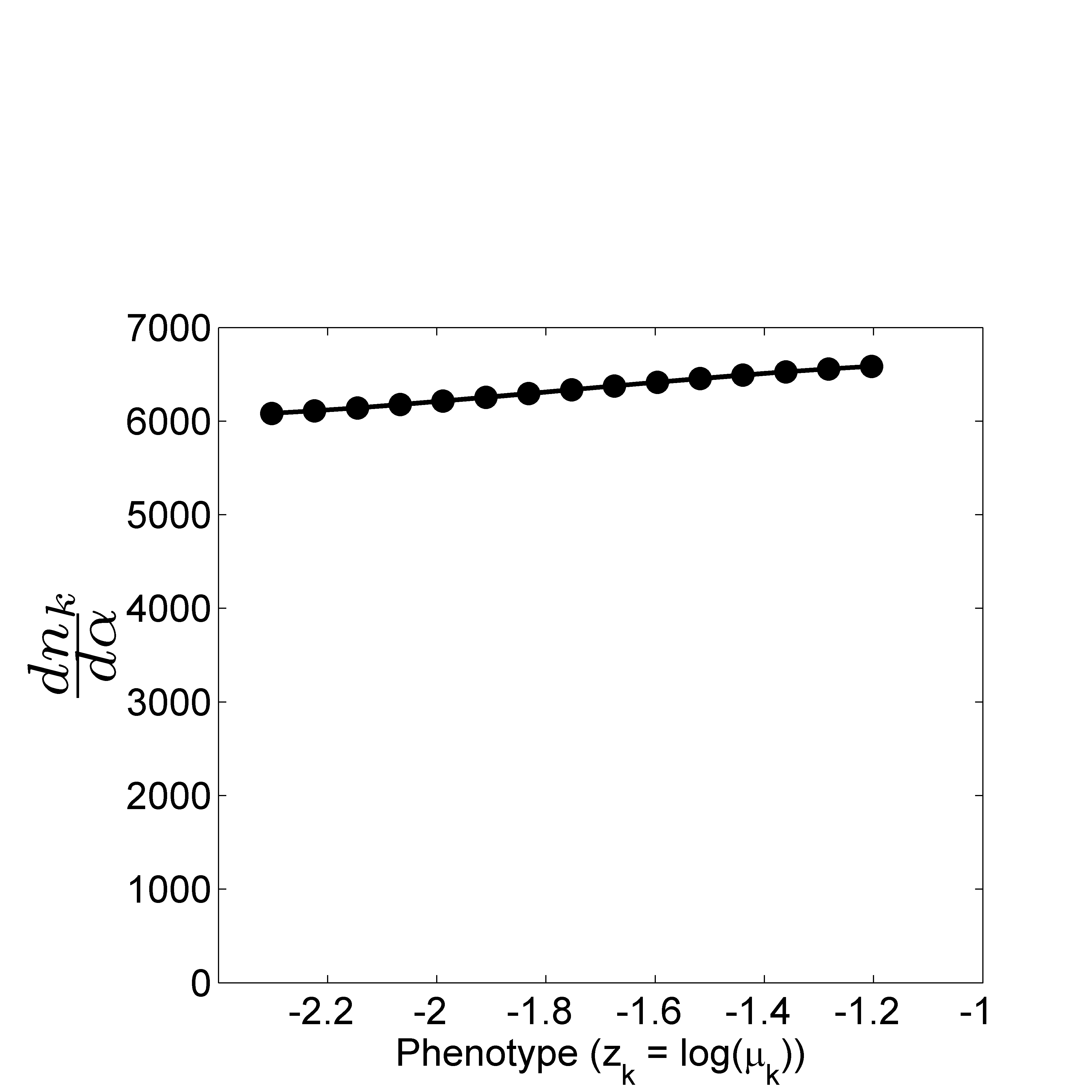

Supplement: Figure S10 — The response of a fixpoint community to a perturbation in α. The plot shows the derivative dnk/dα (evaluated at the fixpoint) for all species k. The system responds very sensitively to changes in α (dnk/dα∼6000), but the response shows little variation between species. Parameters as in Figures 3 and 4. (TIF) [file pcbi.1002017.s010.tif]
